# Simple but not simpler: a systematic review of Markov models for economic evaluation of cervical cancer screening

**DOI:** 10.6061/clinics/2018/e385

**Published:** 2018-06-29

**Authors:** Juliana Yukari Kodaira Viscondi, Christine Grutzmann Faustino, Alessandro Gonçalves Campolina, Alexander Itria, Patricia Coelho de Soárez

**Affiliations:** I Departamento de Medicina Preventiva, Faculdade de Medicina FMUSP, Universidade de Sao Paulo, Sao Paulo, SP, BR; II Instituto do Cancer do Estado de Sao Paulo (ICESP), Hospital das Clinicas HCFMUSP, Faculdade de Medicina, Universidade de Sao Paulo, Sao Paulo, SP, BR; III Instituto de Patologia Tropical e Saude Publica, Departamento de Saude Coletiva, Nucleo de Economia e Avaliacoes da Saude, Instituto de Avaliacao de Tecnologia em Saude, Universidade Federal de Goias, Goias, GO, BR

**Keywords:** Uterine Cervical Neoplasms, Mass Screening, Decision Modeling, Markov Chains, Cost-benefit Analysis

## Abstract

The aim of this study was to critically evaluate the quality of the models used in economic evaluations of screening strategies for cervical cancer prevention. We systematically searched multiple databases, selecting model-based full economic evaluations (cost-effectiveness analyses, cost-utility analyses, and cost-benefit analyses) of cervical cancer screening strategies. Two independent reviewers screened articles for relevance and performed data extraction. Methodological assessment of the quality of the models utilized formal checklists, and a qualitative narrative synthesis was performed. Thirty-eight articles were reviewed. The majority of the studies were conducted in high-income countries (82%, n=31). The Pap test was the most used screening strategy investigated, which was present in 86% (n=33) of the studies. Half of the studies (n=19) used a previously published Markov model. The deterministic sensitivity analysis was performed in 92% (n=35) of the studies. The mean number of properly reported checklist items was 9 out of the maximum possible 18. Items that were better reported included the statement of decision problem, the description of the strategies/comparators, the statement of time horizon, and information regarding the disease states. Compliance with some items of the checklist was poor. The Markov models for economic evaluation of screening strategies for cervical cancer varied in quality. The following points require improvement: 1) assessment of methodological, structural, heterogeneity, and parameter uncertainties; 2) model type and cycle length justification; 3) methods to account for heterogeneity; and 4) report of consistency evaluation (through calibration and validation methods).

## INTRODUCTION

Cervical cancer continues to be an important public health problem, with an estimated 266,000 deaths from cervical cancer worldwide in 2012 (approximately 87% of cervical cancer deaths occur in less developed regions) 1. Screening programs have reduced the incidence and mortality of cervical cancer. However, substantial costs are involved in providing the infrastructure, training the manpower, buying consumables, elaborating surveillance mechanisms, and treating and following up with patients 2. Therefore, successful programs will require using evidence-based, cost-effective approaches and strengthening national health systems 3.

Decision-analytic modeling (DAM) has increasingly been used to assess cancer prevention and control strategies in terms of their cost-effectiveness and to inform public policies. DAM supports decision makers in making choices related to the evaluated screening strategies for cervical cancer options.

Cervical screening models vary considerably in their degree of complexity. The Markov model is the most common model used to simulate the natural history of progression to cervical pre-neoplastic and neoplastic disease. This popularity is likely due to the apparent simplicity of its implementation and use.

Previous reviews 4-8 have specifically discussed the use of DAM to evaluate the cost effectiveness of cervical cancer screening, and others have discussed models that also evaluate the impact of human papillomavirus (HPV) vaccination on screening programs. However, none of these reviews critically evaluated the quality of the Markov models used in economic evaluations of screening strategies for cervical cancer using formal checklists. These instruments may identify flaws that influence the cost-effectiveness results 9. Thus, critical evaluation can confirm the credibility and reliability of the results being used by decision makers 10.

The aim of this review, which was performed as part of a health technology assessment project funded by the Brazilian Public Health System, was to provide an overview of the quality of Markov models for economic evaluation of screening strategies for cervical cancer prevention. We identify some of the most important methodological issues, reflect on the reasons for the poor report and discuss implications for research standards.

## MATERIALS AND METHODS

### Protocol and registration

This methodological systematic review was conducted based on the Centre for Reviews and Dissemination (CRD) guidelines 9 and reported according to the Preferred Reporting Items for Systematic Reviews and Meta-Analyses (PRISMA) checklist 11. A protocol was developed prior to the initiation of this review but was not registered with International Prospective Register of Systematic Reviews (PROSPERO) because this review does not contain direct patient or clinically relevant outcomes.

### Eligibility criteria

Studies were included if they reported on the use of a Markov model to evaluate the costs and health outcomes of cervical cancer screening. Eligibility criteria were defined based on the components of the PICOS approach:

**P**articipants: Markov model for economic evaluation of cervical cancer screening.

**I**ntervention: Cervical cancer screening in settings with or without an HPV immunization program.

**C**omparators: Screening tests: Papanicolaou smear (Pap test), liquid-based cytology (LBC), hybrid capture (HC2), HPV-DNA, visual inspection with acetic acid (VIA), visual inspection with Lugol’s iodine (VILI), and speculoscopy.

**O**utcome: Incremental cost-effectiveness ratio (ICER).

**S**tudy design: Model-based full economic evaluations (cost-effectiveness analyses, cost-utility analyses, and cost-benefit analyses).

This review included only English, Spanish, and German language publications. Editorials, abstracts of congress, review studies, studies that did not compare screening strategies in terms of costs and health consequences, and studies that exclusively analyzed vaccination strategies were excluded.

### Electronic search

An electronic search was performed in the following databases: MEDLINE via PubMed (1946 to August (week 2) 2016), the NHS EED National Health Service Economic Evaluation Database (NHS EED) of the Centre for Reviews and Dissemination (CRD) (1994 to August week 2, 2016), EMBASE (1974 to August week 2, 2016) and Web of Science (1900 to August (week 2) 2016). The search included terms used in previous reviews 4,7,12,13 and relevant studies 14-23: “Markov model” AND (“Uterine Cervical Neoplasms” OR “Cervical Intraepithelial Neoplasia” OR “Squamous Intraepithelial Lesions of the Cervix”) AND “Human Papilloma Virus” AND “Screening” AND “Costs and Cost Analysis”. The electronic search strategies created specifically for each database are provided in Appendix 1.

### Searching other sources

Additional relevant studies were identified by assessing the reference lists of major publications on the subject and the references of studies identified by electronic databases.

### Study selection

This review included only Markov model-based full economic evaluations of cervical cancer screening in settings with or without an HPV immunization program. Two independent reviewers (JYKV and CGF) screened the titles and abstracts of the identified studies and selected them using specific inclusion and exclusion criteria. Any disagreements during this process were resolved by discussion or by a third reviewer (PCS).

### Data collection process

Two reviewers (JYKV and CGF) independently extracted the data into a Microsoft Excel 2016 spreadsheet form tailored to this project. The data collection form was based on a prior publication 4 and was piloted in five studies.

The following data were extracted from all studies:

1. General study characteristics: authors, year of publication, country where the analysis was performed, screening tests for cervical cancer, target population, study type (cost-effectiveness analyses, cost-utility analyses), currency, year of reported costs, ICER, funding sources, conflicts of interest, health outcomes perspective and time horizon of analysis, cost-effectiveness thresholds, and HPV immunization program in place.
2. Model characteristics: use of own model, graphical representation, number of health states, cycle length, software used, calibration of parameters, model validation and types of sensitivity analysis.

### Summary measures conversions

To enable comparisons across studies conducted in different countries and years and account for the effects of inflation over the designated period, the summary measures (ICERs) were updated to the year 2015. When the year of reported costs was not specified, the article publication year was used. Local currencies were initially inflated to 2015 values using specific consumer price indexes 24,25 and then converted into 2015 international dollars (I$) using purchasing power parity conversions provided by the World Bank (http://data.worldbank.org/indicator/PA.NUS.PPP) 26.

### Quality assessment

We evaluated the reporting quality of the structuring and development of Markov models using items of the framework for quality assessment of DAM 27 and a previously described instrument 28. The adapted checklist is an 18-item measure of the overall quality assessment of a DAM and contains three dimensions: 1) structure, 2) data, and 3) consistency (see Appendix 2). We chose these instruments as they are widely accepted as a scientific standard for the reporting of DAM studies, and they can be applied to quality assessment of DAMs for health technology assessment (HTA). The response options for each item include ‘yes’, ‘no’ and ‘not applicable’. Each reviewed study was evaluated individually, and we counted each properly reported item (answer = ‘yes’) and summed responses based on a maximum possible count of 18.

### Synthesis of results

The more relevant results were summarized as a narrative synthesis. The study characteristics are presented in tables and figures. Due to study heterogeneity, meta-analysis or statistical pooling of the extracted summary measure (ICER) was not performed, given that this was neither feasible nor meaningful 29.

## RESULTS

### Search results

**Figure 1:**
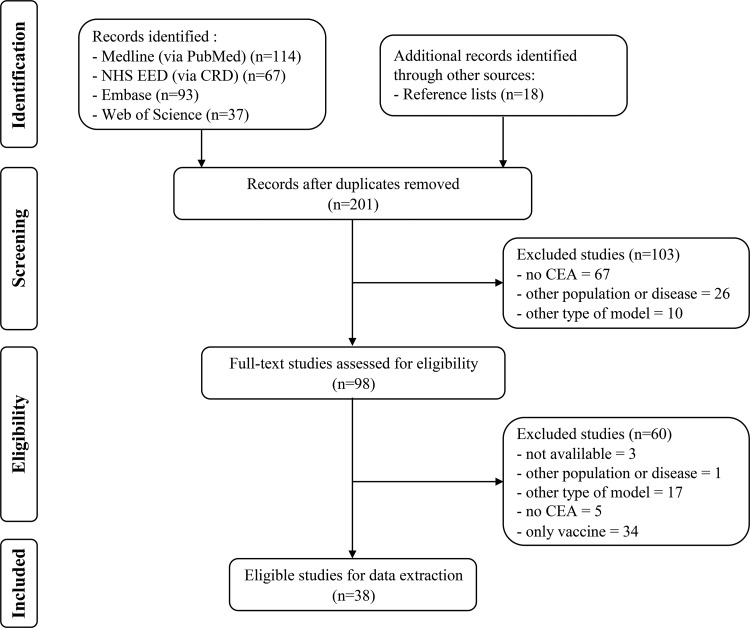
Flowchart of systematic review selection process. HEE: health economic evaluation

After the removal of duplicates, a total of 201 potentially relevant articles were identified. After assessment of the eligibility criteria, 38 studies 30-66 met these review inclusion criteria. Figure 1 presents the flowchart of the selection process.

### Study characteristics

Table 1 presents the economic evaluation of included Markov model-based studies. The majority of the studies were conducted in high-income countries (82%, n=31). Greater than half of the studies were set in three high-income countries (USA=13, GBR=5, and CAN=3). Sixteen percent (n=6) of these studies were conducted in upper-middle income countries, and only one study included lower-middle and low-income countries 48.

The Pap test was the most commonly used screening strategy investigated and was employed in 86% (n=33) of the studies. The LBC, HC2 and HPV-DNA test were employed in 34% (n=13), 29% (n=11) and 24% (n=9) of the studies, respectively. Combined tests, such as Pap + HC2, Pap + HPV-DNA, Pap + speculum and HC2 + cytology, were employed in 26% (n=10) of the studies. Other technologies, such as VIA, VILI and self-collection, were also investigated (16%, n=6). Thirteen studies (34%) considered the effect of an HPV immunization program on the analysis.

The majority of the studies (53%, n=20) were cost-effectiveness analyses, followed by cost-utility analyses (34%, n=13) and a combination of both (13%, n=5). The lowest ICER estimate (I$156.91) was obtained in the African region 48, and the highest (I$1,173,080.66) was noted in Taiwan 46. Most of the calculated ICERs (67%, n=24) could be considered cost-effective strategies.

**Figure 2:**
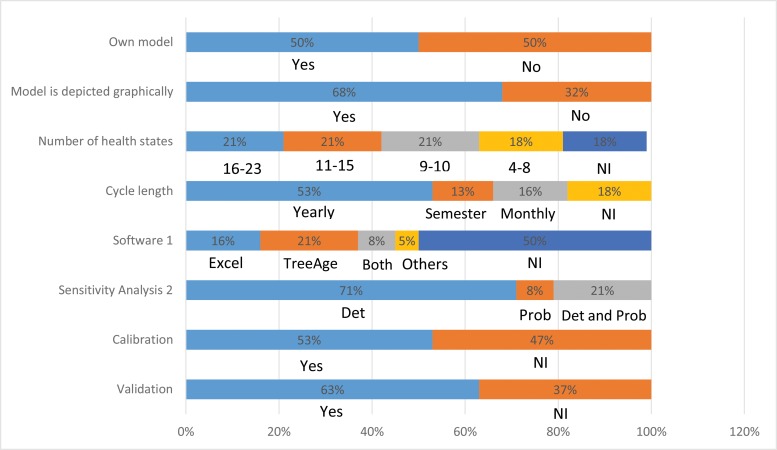
Decision-model characteristics of included studies. ^1^ Software: Others = WHO PopMod or C++ Program. ^2^ Sensitivity analysis: Det = deterministic, Prob = probabilistic. NI = not informed.

Half of the studies (n=19) used a previously published Markov model. In particular, five studies 36,43,49,51,66 used the model developed by Myers et al. 67. A graphical representation was presented in 68% (n=26) of the studies. The number of health states considered when stated (n=31, 82%) ranged from 4 to 23 states (mean of 12). Among the studies that reported the duration of the Markovian cycle used (n=31, 82%), the majority (n=20, 65%) considered annual cycles. Among the studies that reported (n=19, 50%) the use of some software, most studies (n=11, 58%) used TreeAge (TreeAge Software Inc., Williamstown, MA), whereas Excel (Microsoft Corp., Redmond, WA) was used by 47% (n=9) of the studies. One study used software developed by the WHO, PopMod 68, and one study implemented the model using the C ++ programming language 35 (Figure 2).

Deterministic sensitivity analysis was performed in 92% (n=35) of the studies, of which 23% (n=8) also performed probabilistic analysis. The validation of the model was informed by 24 (63%) studies, whereas 53% (n=20) of the studies mentioned that model parameters were calibrated (Figure 2).

**Figure 3:**
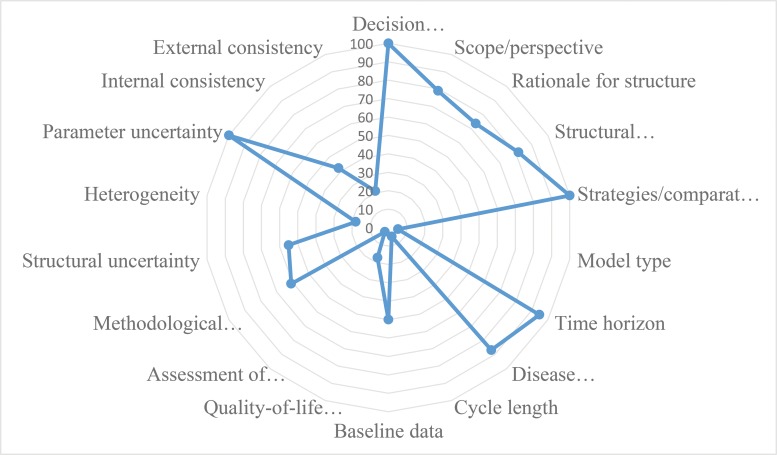
Proportion of economic evaluation studies adequately reporting checklist items (n=38).

Figure 3 presents the proportion of economic evaluation studies (n=38) that properly complied with the 18 items of the checklist domains. The detailed assessment is reported in Appendix 3. The mean number of properly reported checklist items was 9 (SD 2.0) out of the maximum possible 18. Items that were better reported than others were the statement of decision problem (item 1, 100%), the description of the strategies/comparators (item 5, 100%), the statement of the time horizon (item 7, 95%) and informing the disease states (item 8, 87%). Only one study simultaneously assessed the methodological, structural, heterogeneity, and parameter uncertainties (item 12) 61. Compliance was poor for the assessment of structural uncertainty (55%, n=21) and extremely poor for the justification of model type (5%, n=2), cycle length (5%, n=2), assessment of heterogeneity (18%, n=4), the appropriateness of utilities (17%, n=4), and assessment of external consistency (21%, n=8).

## DISCUSSION

This systematic review was the first study to comprehensively assess the methodological quality of the models of previously published studies using items of formal checklists. We evaluated 38 decision-analytic cost-effectiveness models, and the results demonstrated poor compliance with these checklists.

As noted in a previous review 12, only one study has been conducted in lower-middle and low-income countries 48, which exhibit the greatest cervical cancer burden. Approximately 84% of cervical cancer cases occur in less developed countries, with the highest incidences of cervical cancer noted in Africa, Latin America and the Caribbean. This finding reflects a lack of technical expertise and shortage of trained health economists in these regions. This finding also highlights the importance of local studies and enforces the need for strengthening the local modeling capacity.

### Model structure

Half of the included studies (n=19) used a previously published Markov model. Only two studies justified the choice of model type 43,54, and the overwhelming majority did not provide reasons or explain why the use of a Markov model was appropriate. The choice of model type should be appropriate for the problem. In the case of cervical cancer, a Markov model may be suitable if the objective of the study is to assess alternative screening strategies in a setting in which disease prevalence is constant. The Markov model will simulate disease progression for a particular cohort of patients, assigning a probability of progression and regression between each of the classifications of dysplasia and invasive cancer 69. One limitation of the closed population model (such as a Markov cohort model) is that it may predict an increased cancer incidence compared with an open model. If the analysis incorporates the effect of an HPV immunization program, the ideal model would be a dynamic model that follows an entire population, allowing for evaluation of the impact of herd immunity (i.e., indirect protection of susceptible individuals by a significant proportion of immune individuals in the population) 69. Thirteen studies 36,38,45-48,50,55,57,59,62,63,66 reported that the effect of an HPV immunization program was considered in the analysis but did not explain how herd immunity was incorporated using a static cohort model.

The Markov model can be more transparent and easy to understand and provides more conservative estimates than dynamic models. In contrast, because the latter model type allows for the inclusion of more detail, it can generate several uncertainties in the evaluation process in addition to requiring more input and computational resources that may not be available in all settings. The direct and indirect effects of vaccination may not be observed in surveillance data for many years. Thus, although dynamic models are still developed by a small group of modelers 70, the development of these models will become increasingly important to explore the impact on screening as the first vaccinated cohorts approach the age of cervical cancer screening 12. Previous studies have reported an increased screening rate among vaccinated women and the lowest proportion of cervical abnormalities compared with those not vaccinated 71,72. Future model-based economic evaluations will need to take into account the continuum interaction between screening and vaccination to predict the effects of vaccination on screening programs 6.

Only two studies justified the choice of cycle length 56,64. The cycle length should reflect the clinical problem and be the shortest interval at which the pathologies and/or diagnosis typically occurs 73, and its justification should be based on the natural history of the disease 74. In the case of cervical cancer, often the only source of information regarding cases is the clinical examination results. However, this information may be under-reported given that HPV infections and precursor lesions may regress in less than a year 75 and screening is typically performed annually. Therefore, ideally, the definition of the cycle should not be based on the intervals between exams 74. However, occasionally, these are the only available data. The other option would be to use data from another setting, and both approaches would impact the analysis results.

### Model data

Although half of the included studies presented the transition probabilities, none of them explained how the probabilities were calculated or whether the cycle correction was used. Concern has been raised in the DAM literature regarding confusion about the appropriate use of rates and probabilities. Depending on the model, this misconception may introduce important errors, impacting the validity of the model results 76,77. Various approaches can be used to estimate transition probabilities for the natural history of cervical cancer in Markov models, including a literature review of HPV and cervical intraepithelial neoplasia (CIN) progression and regression rates, data from observational studies, and fitting approaches 78. Although some relevant publications exist, no formal guidelines are available for the estimation of transition probabilities for use in Markov models 79. The understanding of the difference between rates and probabilities and how to transform them correctly is essential for those developing Markov models.

According to international guidelines, if health benefits are measured through utility measures, the methods used (e.g., time trade-off, standard gamble, specific questionnaires) and the subjects in whom the assessments were performed (e.g., patients, members of the general public, health professionals) need to be reported 80,81. Only 17% of the reviewed studies reported the applied instruments, methods of measurement and the sources of utilities employed. Inadequate reporting of utility measurement methods leads first to difficulties in comparing different assessments, given that discrepancies between these measures using different measurement instruments and methods were previously observed in other studies 82-84. In addition, in relation to the lack of reporting of sources of utility measures (populations used to derive these measures), if the ultimate objective of the evaluation is to influence the allocation of resources to decisions based on social interests, it would be important that health state evaluations were based on utility weights representative of the preferences of the general population 85.

Specifically in relation to economic evaluations of cervical cancer screening, differences in utility values for CIN lesions, presence of cervical cancer and genital warts may partially explain the differences in the analysis results. In addition, considering the limited data available on the utility values associated with these states 7, it is fundamental that sensitivity analyses performed in future studies consider a wide range of variation, including all plausible utility values.

### Uncertainty

Uncertainty is present in all HTA models 74. DMA researchers distinguish among parameter, structural and methodological uncertainties, all of which require assessment 27. Parameter uncertainty can be addressed by deterministic or probabilistic sensitivity analysis. Structural uncertainty can be managed through alternative model structures, which involves re-running the model under alternative structural assumptions and presenting the results of each scenario. Methodological uncertainty can be addressed with a similar method. Only one study simultaneously assessed methodological, structural, heterogeneity, and parameter uncertainties 61. Approximately half of the included studies failed to account for structural uncertainty, reflecting the gap between guidelines and applied research. This finding was also highlighted in a previous review 28, where many published models failed to account correctly for the major sources of uncertainty, particularly structural uncertainty. Most studies (92%) addressed only parameter uncertainty through deterministic sensitivity analysis. In addition to the standard considerations of uncertainty about parameter estimates, it is important to assess the implications of model uncertainty on results 28.

Most models (89%, n=34) simulated aggregate groups of women at risk of cervical cancer over time without accounting for other aspects of population heterogeneity in screening behavior. Heterogeneity (i.e., the extent to which variability between patients can be explained as a function of their characteristics) 86 reflects differences in outcomes that may in principle be explained by variations among subgroups of patients, including characteristics such as age, sex, level of risk and severity of the disease, or the relative effects of treatment 87. Given the natural history of cervical cancer, women less than 30 years of age have more HPV infections than older women, while older women may experience the progression of this virus 116-fold more frequently than younger women. Therefore, HPV-DNA screening after the age of 30 years seems to be more effective than before the age of 30 88. Thus, not considering "heterogeneity" during the analysis, which could be performed by executing the model for different subgroups of patients, may lead to errors in the results obtained 89. To capture heterogeneity in screening and vaccination behavior, it would be ideal to use individual-based models (microsimulation).

### Model consistency

Model consistency refers to the quality of the model overall. This parameter tests the internal logic of the modeling practice, changing model inputs and examining the direction of results (internal consistency). Model consistency also compares the model’s result with the best available evidence or with the results of previously developed models (external consistency, also known as calibration). For instance, the model consistency of cervical precancerous lesions predicted by cytology can be compared with observed CIN-related outcomes. However, it is generally not clear whether these outcomes are predicted by cytological results or histologically confirmed lesions 8. Only 8 studies (21%) reported the use of some calibration method. This low value can be explained by the lack of standards in calibrating disease models in economic evaluation, especially cancer screening models 90,91. There is no consensus in the literature regarding an acceptable minimum specification for the fitting targets that should be reported 78. Another potential barrier to calibration is insufficient local data to estimate parameters associated with organized screening.

The Markov models for economic evaluation of screening strategies for cervical cancer varied in quality. Items that were generally well reported were the statement of the decision problem, the description of the strategies/comparators, the statement of time horizon, and informing disease states. One limitation of the present study is that most models did not adequately assess methodological, structural, heterogeneity, and parameter uncertainties. Moreover, the minority justified the model type and cycle length, assessed heterogeneity and the appropriateness of utilities, and evaluated external consistency. Future studies should evaluate the appropriateness of the different methods to account for uncertainty (through sensitivity analysis and alternative model structures), heterogeneity, consistency (through calibration and validation techniques), and the relevance of reporting guidelines for Markov models to improve their transparency.

## AUTHOR CONTRIBUTIONS

Viscondi JY and De Soárez PC designed the research. Viscondi JY, Faustino CG and Itria A performed the research. Viscondi JY, Faustino CG, Campolina AG and De Soárez PC analyzed the data. Viscondi JY, Campolina AG and De Soárez PC wrote the manuscript.

## Figures and Tables

**Figure:**
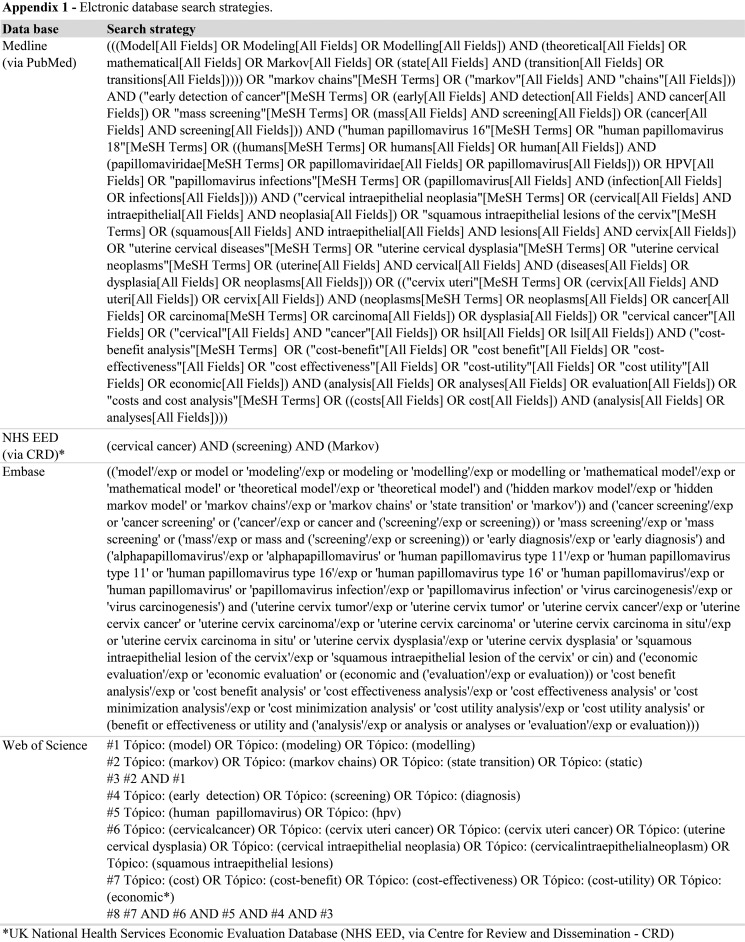


**Figure:**
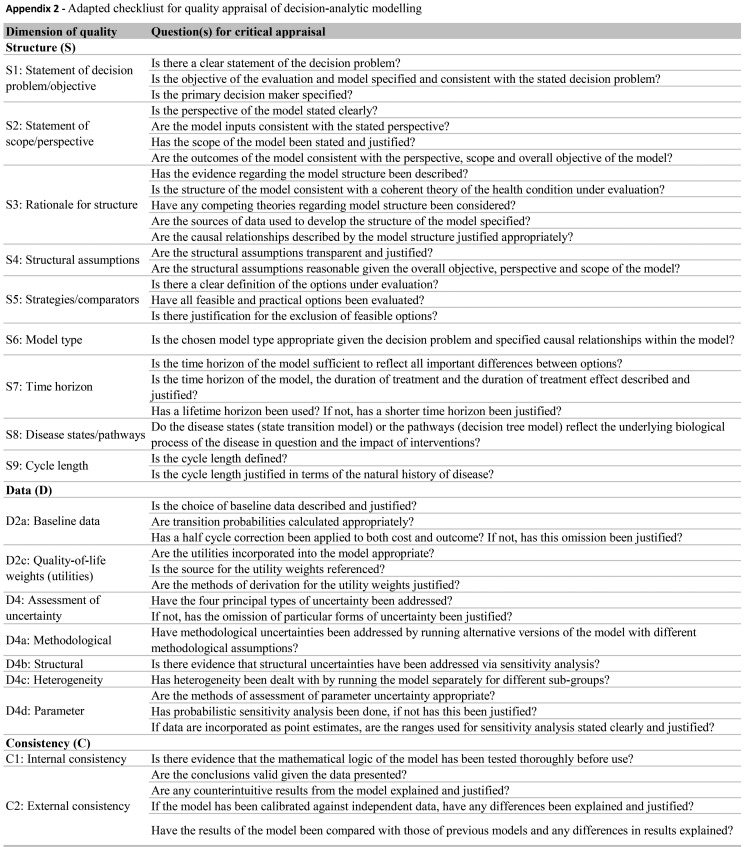


**Figure:**
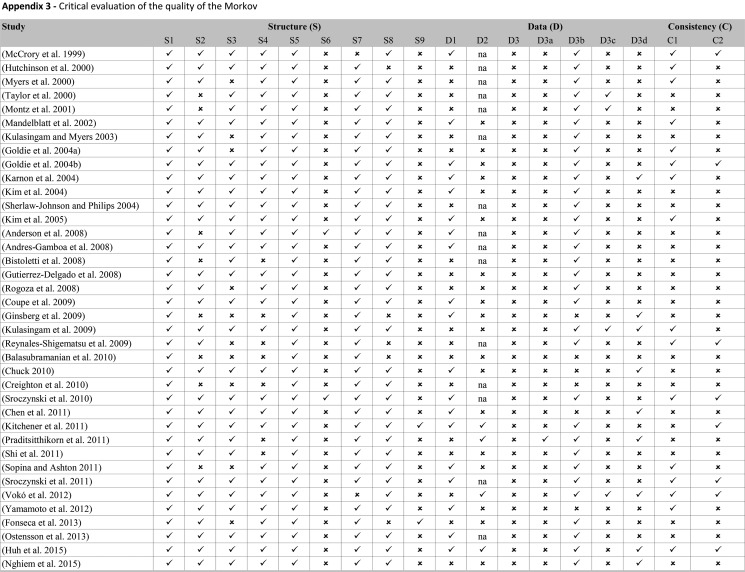


**Table 1:** Characteristics of the economic evaluation of the included Markov model-based studies.

Study	Country ^1^	Screening tests ^2^	Target pop ^3^	Study type ^4^	Currency (year) ^5^	ICER (I$) ^6^
(McCrory et al. 1999)	USA	PAP, NewCyto	15-85 years	CEA	USD (1997)	32,503.08/LY
(Hutchinson et al. 2000)	USA	PAP, AutoPap, LBC	20-65 years	CEA	USD (1997)	73,837.07/LY
(Myers et al. 2000)	USA	PAP, NewCyto	15-85 years	CEA	USD (1997)	4,310.60/LY
(Taylor et al. 2000)	USA	PAP, PAP+speculoscopy	18-65 years	CEA	USD (2001)	not calculated
(Montz et al. 2001)	USA	PAP, LBC	20-80 years	CEA	USD (1997)	26,532.61/LY
(Mandelblatt et al. 2002)	USA	PAP, LBC, HC2	≥20 years	CUA	USD (2000)	96,826.00/QALY
(Kulasingam and Myers 2003)	USA	PAP	12-85 years	CEA	USD (2001)	60,076.00/LY
(Goldie et al. 2004a)	USA	PAP, LBC, HC2, HC2+Cyto	≥30 years	CEA	USD (2001)	41,488.03/LY
(Goldie et al. 2004b)	USA	PAP, LBC, HC2	≥12 years	CUA	USD (2002)	77,073.34/QALY
(Karnon et al. 2004)	GBR	PAP, LBC	15-95 years	CEA	GBP (NI)	16,782.16/LY
(Kim et al. 2004)	HKG	PAP, LBC	≥15 years	CEA	USD (2000)	12,821.96/LY
(Sherlaw-Johnson and Philips 2004)	GBR	PAP, LBC, HPV test	≥15 years	CEA	GBP (2001)	6,010.34/LY
(Kim et al. 2005)	GBR, NLD, FRA, ITA	PAP, HC2, PAP+HC2	NI	CEA	USD (2004)	23,590.95 - 106,985.77/LY
(Anderson et al. 2008)	AUS	PAP	15-85 years	CEA	USD (NI)	252,485.84/LY
(Andres-Gamboa et al. 2008)	COL	PAP, HPV test	15-76 years	CEA	USD (2006)	262,302.32/LY
(Bistoletti et al. 2008)	SWE	PAP, PAP+HPV test	≥32 years	CEA	USD (2005)	7,132.05/LY
(Gutierrez-Delgado et al. 2008)	MEX	PAP, HC2, PAP+HC2	12-64 years	CUA	MXN (2006)	209,410.00/DALY
(Rogoza et al. 2008)	CAN, NLD, TWN, GBR, USA	PAP	≥12 years	CEA and CUA	CAD,EUR,NTD,GBP,USD (2006)	9,203.22 - 1,173,080.66/QALY
(Coupe et al. 2009)	NLD	PAP, HPV test	12-100 years	CUA	EUR (2006)	11,810.39/QALY
(Ginsberg et al. 2009)	WHO 14 regions	PAP, HPV test, PAP+HPV test, VIA	NI	CUA	I (2000)	156.91 - 63,616.09/DALY
(Kulasingam et al. 2009)	CAN	PAP, HC2, PAP+HC2	NI	CEA and CUA	CAD (2006)	7,060.14/LY
(Reynales-Shigematsu et al. 2009)	MEX	PAP	12-85 years	CEA	USD (2004)	2,075.00/LY
(Balasubramanian et al. 2010)	USA	LBC, HPV test, Self-colletion	12-85 years	CUA	USD (2007)	15,142.00/QALY
(Chuck 2010)	CAN	PAP, LBC, HC2	12-80 years	CUA	CAD (2007)	16,528.54/QALY
(Creighton et al. 2010)	AUS	PAP	10-84 years	CEA	AUD (NI)	30,583.80/LY
(Sroczynski et al. 2010)	DEU	PAP, PAP+HC2	≥15 years	CEA	EUR (2007)	107,285.43/LY
(Chen et al. 2011)	TWN	PAP, HPV test	NI	CEA	USD (NI)	64,249.86/LY
(Kitchener et al. 2011)	GBR	LBC, HC2, Self-colletion	10-84 years	CEA and CUA	GBP (2007)	49,193.51/LY
(Praditsitthikorn et al. 2011)	THA	PAP, VIA	≥15 years	CUA	THB (2007)	543,574.08/QALY
(Shi et al. 2011)	CHN Rural	VIA, VIA/VILI, HPV test	NI	CEA and CUA	USD (2009)	30,206.17/LY
(Sopina and Ashton 2011)	NZL	LBC, HPV test	12-85 years	CEA and CUA	NZD (2009)	9,164.45/QALY
(Sroczynski et al. 2011)	DEU	PAP, HC2, PAP+HC2	≥15 years	CEA	EUR (2007)	177,473.23/LY
(Vokó et al. 2012)	HUN	PAP	≥25 years	CUA	HUF (NI)	33,339.15/QALY
(Yamamoto et al. 2012)	JPN	PAP	≥11 years	CUA	JPY (2010)	28,865.17/QALY
(Fonseca et al. 2013)	BRA Amazon	PAP	≥12 years	CUA	USD (NI)	1,341.06/QALY
(Ostensson et al. 2013)	SWE	PAP, HPV test	≥15 years	CEA	EUR (2011)	8,795.19/LY
(Huh et al. 2015)	USA	PAP, HPV test, PAP+HPV test	30-70 years	CUA	USD (2013)	7,800.62/QALY
(Nghiem et al. 2015)	USA	LBC, HPV test	≥12 years	CUA	USD (2012)	18,854.49/QALY

1 Countries classified according to the list of country names and 3-letter codes abbreviations by the United Nations (http://unstats.un.org/unsd/methods/m49/m49alpha.htm).

2 Screening tests: PAP = conventional cytological test also known as Papanicolaou (pap) smear test; New Cyto = hypothetical new cytological test; AutoPap = automated read cytological test; LBC = liquid-based cytology test; HC2 = Digene high-risk HPV Hybrid Capture^©^ 2 test (Qiagen); HPVtest = Human Papillomavirus (HPV) DNA detection with genotyping high-risk types by polymerase chain reaction (PCR); Self-collection = high-risk HPV DNA testing of self-collected vaginal samples; VIA = visual inspection with acetic acid; VIA/VILI = VIA in combination with Lugol’s iodine.

3 Target population: women within the age range indicated.

4 Economic study type: CEA = cost-effectiveness analysis; CUA = cost-utility analysis.

5 Currencies classified according to the International Organization for Standardization, ISO 4217:2015 (http://www.iso.org/iso/home/standards/currency_codes.htm).

6 ICER = incremental cost-effectiveness ratio; I$: Geary-Khamis dollar, more commonly known as the international dollar; LY = life years; QALY = quality-adjusted life years; DALY = disability-adjusted life years; NI = Not informed.
